# Comprehensive profile and natural history of pediatric patients with spinal muscular atrophy: A large retrospective study from China

**DOI:** 10.3389/fneur.2022.1038012

**Published:** 2022-12-20

**Authors:** Chaoping Hu, Xihua Li, Yiyun Shi, Xiaomei Zhu, Lei Zhao, Wenhui Li, Shuizhen Zhou, Yi Wang

**Affiliations:** Department of Neurology, Children's Hospital of Fudan University, Shanghai, China

**Keywords:** spinal muscular atrophy, survival motor neuron, survival analysis, pattern, motor deterioration

## Abstract

**Background:**

There is a large population of people with spinal muscular atrophy (SMA) in China, and new disease-modifying therapies have become available recently. However, comprehensive data on the management and profile of treatment-naive SMA patients in China are still lacking.

**Methods:**

As a retrospective study, a large cohort of treatment-naive patients with clinical and genetic diagnoses of 5q SMA were enrolled, ranging from neonatal to 18 years old, from the Neurology Department of Children's Hospital of Fudan University between January 2013 and December 2020. The data regarding their clinical presentations, genetic defects, motor function assessment results, and follow ups were reviewed.

**Results:**

We enrolled 392 SMA patients (male: female = 189: 203): 1a = 46, 1b = 44, 1c = 31, 2a = 119, 2b = 56, 3a = 52, 3b = 14, from 27 of the 34 administrative districts in China, and 389 patients harbored homozygous deletion of exon 7 in the SMN1 gene (99.2%). The median age of onset was 0.08 (range: 0–0.30), 0.25 (0.06–0.60), 0.42 (0.08–1.50), 0.67 (0.07–5.08), 1.0 (0.40–1.83), 1.5 (1.00–3.00), and 4.04 (1.80–12.00) years old for SMA 1a, 1b, 1c, 2a, 2b, 3a, and 3b patients, while the median age of first assessment was 0.25 (0.08–2.60), 0.42 (0.17–1.90), 0.80 (0.17–4.5), 2.50 (0.5–15.83), 2.92 (1.08–13.42), 4.25 (1.58–17.33), and 7.34 (3.67–14.00) years old, respectively. Patients were followed up with for up to 15.8 years. The median event-free survival time was 7 months, 15 months, and indeterminate in SMA 1a, 1b, and 1c patients (*p* < 0.0001), with a better survival situation for higher SMN2 copies (*p* = 0.0171). The median age of sitting loss was 5.75 years and 13.5 years in SMA 2a and 2b (*p* = 0.0214) and that of ambulation loss was 9.0 years and undefined in SMA 3a and 3b (*p* = 0.0072). Cox regression analysis showed that higher SMN2 copies indicated better remaining ambulation in SMA 3. The median time to develop orthopedic deformities was 4.5, 5.2, and 10.1 years in SMAs 1c, 2, and 3, respectively (*p* < 0.0001), with a possible trend of better preservation of joint function for patients under regular rehabilitation (*p* = 0.8668).

**Conclusion:**

Our study elucidated insight into the comprehensive management and profile of different types of SMA patients in China, providing a clinical basis for assessing the efficacy of new therapies.

## Background

Spinal muscular atrophy (SMA) is a neuromuscular disorder caused by homozygous absence by deletion or gene conversion events (90%), hybrid genes (5%), or subtle disease-causing variants (<5%) of the survival motor neuron 1 (SMN1) gene (1, 2). SMA has a panethnic incidence of ~1 in 11,000 live births in Caucasian individuals (3) and the carrier frequency is ~1/42 in the general Chinese population (4, 5). SMA represents a continuous spectrum of phenotypes ranging from variously compromised neonates and infants to adults with minimal manifestations. Age at onset and acquired motor milestones have been used to define at least 4 SMA types.

Recently, new treatment strategies, including nusinersen, risdiplam and onasemnogene abeparvovec, have remarkably improved survival and motor function in the short term as well as changing the disease course in the longer term to varying degrees. However, natural history studies in SMA patients remain crucial, either for the design of clinical trials of advanced treatments or for the expansion of investigation of trajectory and predictors analysis. Over the last two decades, there has been increasing understanding of the natural history of SMA types (6, 7) and the correlation between the severity of the clinical phenotype and the SMN2 copy number (8).

Although motor function decreases rapidly in SMA 1 patients, trajectories of disease progression and survival situation vary among subgroups of SMA type 1. Milder SMA 1 patients acquired motor milestones such as head control, kicking, and hand grasping, while typical SMA 1 patients acquired none. Patients with later onset had longer preservation of motor function (9). In contrast, disease progression of subtypes with early onset and low scores of motor function showed a more rapid and sharper decline in motor function (10, 11). Although survival analysis of SMA type 1 patients with 3 SMN2 copies showed an overall milder phenotype and milder progression than that with 2 SMN2 copies (12), 2 SMN2 copies were found in all three subgroups of SMA type 1 (10). This phenomenon suggests that SMN2 copy number is a strong phenotype modifier, nevertheless not the only one. In one report, the initial TIMPSI score (Test of Infant Motor Performance Screening Items) was inversely associated with the risk of death or persistent ventilation, while no compound muscle action potential (CMAP), weight or CHOP-INTEND was associated with prognosis, even though CMAP decreased rapidly in SMA infants (12).

Motor function and trajectories of progression also vary among SMA types 2 and 3. Motor function, pulmonary function, and muscle strength measures in SMA 2 and 3 remained relatively stable over 12 months (13), but showed slow functional declines when observation periods exceeded 1 year (14). A cross-sectional study showed that whole-body muscle mass, hand muscle compound motor action potentials (CMAPs), and muscle strength were associated with clinical measures of motor function (11). A large cohort study from Poland showed that the mean age of loss of ambulation was 14.0 years in SMA 3 patients, and patients with SMA3a and 3 copies of SMN2 had significantly worse prognosis for loss of ambulation than patients with SMA3b and 4 copies of SMN2, and sex also to some degree affected the course of SMA (15).

Here, we enrolled a large cohort of treatment-naive SMA patients from China and reviewed the clinical presentations, genetic results, and motor function assessments to provide benchmark data on the natural history of SMA and investigate the predictors of prognosis.

## Methods

### Patients

All patients with a clinically and genetically confirmed diagnosis of 5q SMA, in whom data were retrospectively available in the datasets, were considered for inclusion, ranging from neonatal to 18 years old, from the Neurology Department of Children's Hospital of Fudan University between January 2013 and December 2020.

Demographic data were collected, including sex, age upon enrollment, ethnicity, and geographical distribution. Detailed data from the patients' medical files and their parents' interviews was collected and reviewed, including the clinical presentations, genetic results, serum and electrophysiological biomarkers when available. The genetic tests were carried out in several laboratories in China, and the detective methods included multiplex ligation-dependent probe amplification (MLPA), Quantitative Real-time PCR method, CPR-sanger sequencing and next generation sequencing.

If patients had enrolled in a therapeutic clinical trial or started using new drugs including nusinersen, risdiplam or onasemnogene abeparvovec, they were censored at the day of enrollment or the first day of therapy (*n* = 25; 6.4% of included patients). Thus, our analyses are based on treatment-naive patient data.

### Clinical classification and motor function test

Patient characteristics were documented using a combination of parental questionnaires and physical examinations. We distinguished SMA types and subtypes, including SMA 1a−1c, SMA 2a−2b, and SMA 3a−3b, based on age at symptom onset and acquired maximal motor milestones using the SMA classification system (16). Medical Research Council (MRC) scores ranging from 0 to 5 with subscores (e.g., “4+” or “4.5”) were used to test motor function, and the sum of scores of bilateral distal and proximal limbs was calculated. The age of acquirement and loss of main motor milestones, including independent sitting and walking in different SMA subtypes, were reviewed and analyzed in patients when available.

### Statistical analysis

We used all available patient data for analyses, except in cases of significant missing data, as specified. GraphPad Prism software (version 6.01) was used for statistical analysis. For variables distributed in a normal fashion, the mean ± standard deviation was calculated. For not-normally distributed variables, the median (IQR) or median (range) was calculated. Student's *t*-test and the Mann–Whitney *U*-test were performed for normally and not-normally distributed variance of two group comparisons, respectively. Single factor analysis of variance (ANOVA) was performed for multiple group comparisons. The chi-square test or Fisher's exact test was performed for the qualitative variables according to the numbers of samples. Pearson and Spearman correlations were used to demonstrate the interdependence of the examined traits.

Analyses of the change over time in event-free survival, loss of independent sitting and walking, and the occurrence of orthopedic deformities were estimated by the Kaplan–Meier method, and any differences were evaluated with a stratified log-rank test. Multivariable analyses with the Cox proportional hazards model were used to estimate the simultaneous effects of prognostic factors on survival and other outcomes. Interactions with prognostic factors were also examined with the Cox proportional hazards model.

## Results

### Demographic and clinical characteristics

**Figure 1:**
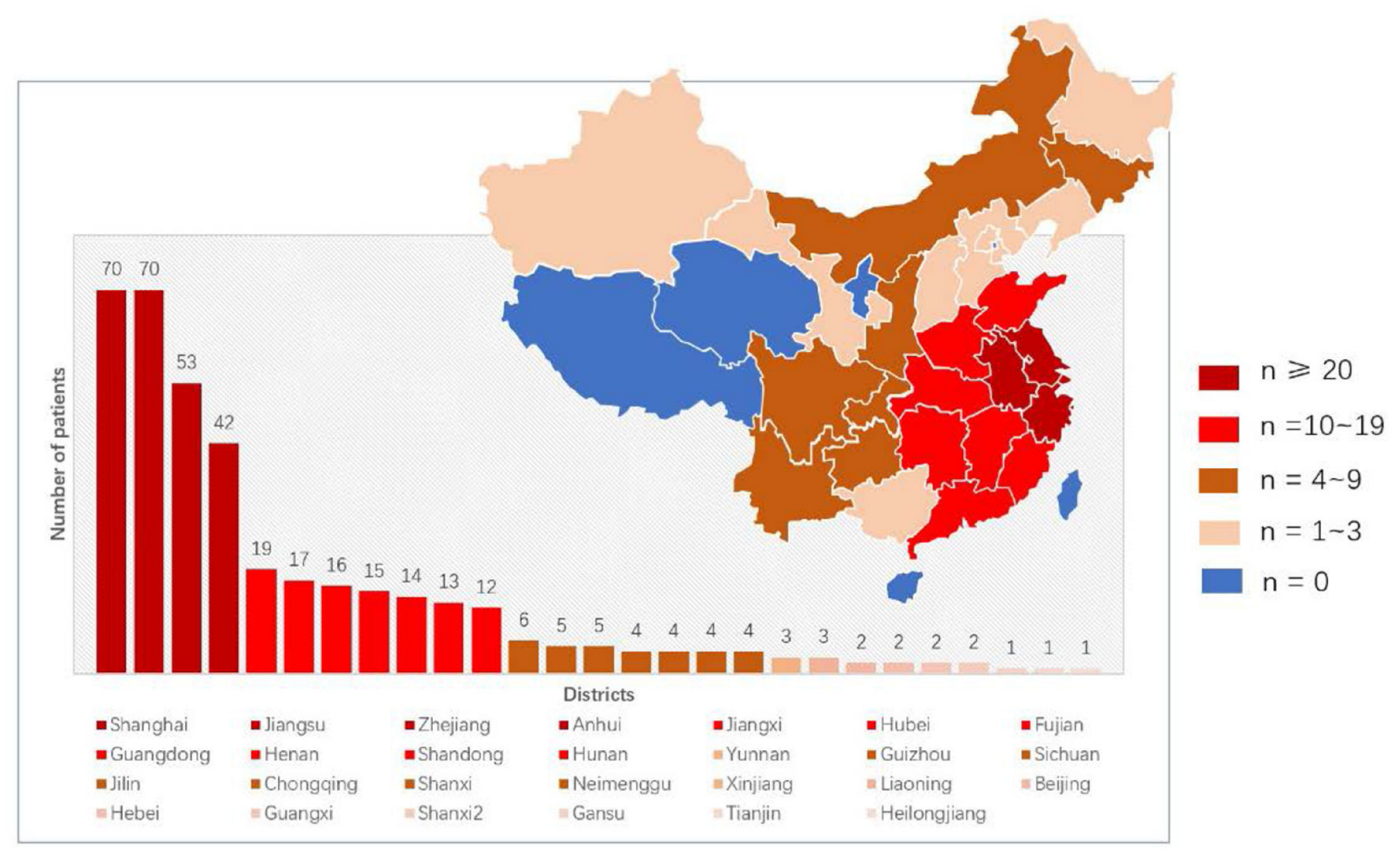
Distribution of 392 SMA patients in our cohort.

We included 392 patients with SMA, 189 boys and 203 girls. They were all of Asian Han nationality. The patients' regions of residence were distributed in 27 of the 34 administrative districts in China (Figure 1).

The cohort comprised 121 cases with SMA type 1 (1a = 46, 1b = 44, 1c = 31), 175 cases with type 2 (2a = 119, 2b = 56), 66 cases with type 3 (3a = 52, 3b = 14), and 30 patients with unknown type. The baseline characteristics of 362 patients are shown in Table 1, including the median age of onset, diagnosis, and first assessment.

**Table 1:** Baseline characteristics and follow-ups since the first assessment of the cohort of 392 SMA patients.

**SMA type**	**Type 1 (*****n*** = **121)**			**Type 2 (*****n*** = **175)**		**Type 3 (*****n*** = **66)**		***P*-value**
	**Type 1a** ** (*n* = 46)**	**Type 1b** ** (*n* = 44)**	**Type 1c ** **(*n* = 31)**	**Type 2a** ** (*n* = 119)**	**Type 2b** ** (*n* = 56)**	**Type 3a** ** (*n* = 52)**	**Type 3b** ** (*n* = 14)**	**/**
Male: Female	24:22	15:29	14:17	69:50	27:29	26:26	7:7	*P* = 0.2377
Family history (*n*, %)	2 (4.3%)	6 (13.6%)	1 (3.2%)	4 (3.4%)	5 (8.9%)	3 (5.8%)	1 (7.1%)	*P* = 0.7574
2 SMN2 copies	19	20	6	8	3	1	1	*P* < 0.0001
3 SMN2 copies	1	6	12	60	33	20	5	
4 SMN2 copies	0	0	1	3	1	4	2	
NA	26	18	12	48	21	27	6	
Age of onset (years): median (range)	0.08 (0–0.30)	0.25 (0.06–0.60)	0.42 (0.08–0.50)	0.67 (0.07–5.08)	1.0 (0.40–1.83)	1.5 (1.00–3.00)	4.04 (1.80–12.00)	*P* < 0.0001
Age of diagnosis (years): median (range)	0.30 (0.08–3.6)	0.40 (0.17–1.5)	0.71 (0.3–6.25)	1.0 (0.50–13.00)	1.5 (0.8–3.67)	2.33 (1.08–13.00)	6.17 (3.00–12.00)	*P* < 0.0001
Interval from onset to diagnosis (years): median (range)	0.17 (0.02–3.5)	0.12 (0.05–1.08)	0.26 (0.02–3.5)	0.41 (0.07–6.25)	0.35 (0.07–2.67)	1.09 (0.08–10.00)	1.04 (0.08–6.28)	*P* < 0.0001
Age of first assessment (years): median (range)	0.25 (0.08–2.60)	0.42 (0.17–1.90)	0.80 (0.17–4.5)	2.50 (0.5–15.83)	2.92 (1.08–13.42)	4.25 (1.58–17.33)	7.34 (3.67–14.00)	*P* < 0.0001
MRC score: mean (SD)	7.4 (2.1)	9.4 (2.4)	10.3 (2.4)	10.0 (2.6)	11.1 (2.5)	13.2 (2.9)	15.8 (1.7)	*P* < 0.0001
CMAP of median nerve (mv): median (range)	0.4 (0.1–0.6)	0.5 (0.2–1.2)	0.8 (0.2–2.2)	1.9 (0.5–3.8)	2.2 (1.1–3.2)	6.1 (5.1–8.2)	5.7 (4.6–8.1)	*P* < 0.0001
CMAP of tibial nerve (mv): median (range)	0.35 (0.01–1.0)	0.40 (0.02–1.2)	0.45 (0.2–3.5)	0.80 (0.6–2.1)	0.96 (0.6–1.3)	3.0 (2.8–16.2)	4.2 (3.9–7.3)	*P* = 0.0008
Creatine kinase (mmol/L): median (range)	163 (42–785)	175 (50–339)	122.5 (46–220)	143.5 (32–366)	183 (89–309)	157 (64–387)	190 (73–663)	*P* = 0.2056
Creatinine (mmol/L): median (range)	13.00 (6–21)	14.00 (3–19)	9.500 (7–16)	13.90 (7–18)	15.50 (12–26)	19.00 (11–29)	28.00 (19–34)	*P* < 0.0001
Follow up time (years): median (range)	0.21 (0.08–3.4)	0.17 (0.07–0.5)	0.63 (0.03–6.67)	2.5 (0.07–15.1)	2.5 (0.02–13.0)	2.4 (0.08–15.8)	4.6 (0.08–8.0)	*P* < 0.0001
Orthopedic deformation: *n* (%)^#^	(22.2%)	3 (8.3%)	5 (19.2%)	46 (48.4%)	23 (44.2%)	16 (41.0%)	2 (20%)	*P* = 0.0272
Feeding problem: *n* (%)	21 (45.7%)^*^	18 (40.9%)^*^	6 (19.4%)^*^	26 (21.8%)^&^	10 (17.2%)^&^	6 (11.5%)^&^	0	*P* < 0.0001
Coughing assistance: *n* (%)	1 (2.2%)	0 (0%)	0 (0%)	2 (1.7%)	0 (0%)	0 (0%)	0 (0%)	*P* > 0.9999
Assisted ventilation: *n* (%)	8 (17.4%)	4 (9.1%)	3 (9.7%)	8 (6.7%)	1 (1.8%)	0 (0%)	0 (0%)	*P* = 0.0244
Pneumonia: *n* (%)	27 (58.7%)	16 (36.3%)	10 (32.3%)	46 (39.3%)	17 (30.9%)	12 (23.1%)	0 (0%)	*P* = 0.0388
Regular rehabilitation: *n* (%)	0 (0%)	5 (11.3%)	4 (12.9%)	39 (32.8%)	16 (28.6%)	11 (21.2%)	3 (21.4%)	*P* < 0.0001
Irregular rehabilitation: *n* (%)	9 (20%)	11 (25.0%)	11 (35.5%)	34 (28.6%)	17 (30.3%)	15 (28.8%)	7 (50.0%)	
No rehabilitation: *n* (%)	37 (80%)	28 (63.6%)	16 (51.6%)	46 (38.7%)	23 (41.1%)	26 (50.0%)	4 (8.6%)	

Orthopedic deformation: *n* (%)

# , indicates the proportion of orthopedic deformities of patients (when available) at the last visit. Feeding problem: *n* (%)

* indicates the portion of patients with feeding difficulty for whom a nasal tube or gastrostomy was necessary, *n* (%)

& indicates the portion of patients with feeding problems for whom a soft or fine and soft diet was necessary.

### Genetic data of 392 SMA patients

All patients were diagnosed with 5q SMA according to molecular tests, consisting of 389 patients with homozygous deletion of exon 7 in the SMN1 gene (389/392, 99.2%), and 3 cases with a point mutation of the SMN1 gene coexisting with a deletion (3/392, 0.8%), including one SMA 1a patient with a splicing mutation (NM_000344.3: c.835-1G>A), one SMA 1c patient with a missense mutation [NM_000344.3: c.835G>C (p.Gly279Arg)], and one SMA 3a patient with a missense mutation [NM_000344.3: c.863G>T (p.Gly279Glufs^*^5)]. Twenty-two patients had a family history of SMA (22/392, 5.6%). Out of 144 families who performed molecular tests by MLPA method, 128 pedigrees showed that both parents were SMA carriers (128/144, 88.9%), 12 pedigrees showed that only one parent carried the mutation, while the other was normal (12/144, 8.3%), and 4 pedigrees revealed that one parent was an SMA carrier, while the other was unavailable (4/144, 2.8%).

**Figure 2:**
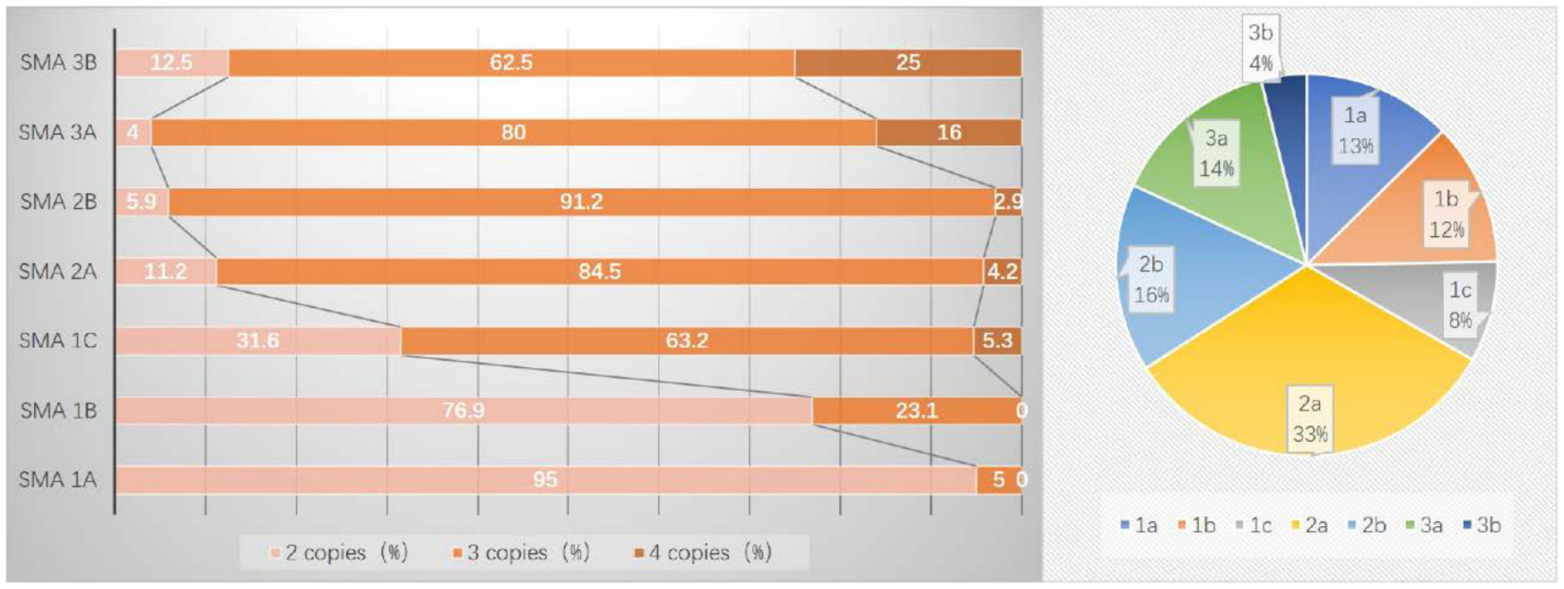
Distribution of SMN2 copy numbers and subtypes in 206 SMA patients.

Data on SMN2 copy numbers were available for 206 SMA patients (Figure 2). The median age of onset was 0.25 (range: 0.04–3.0) years in patients with 2 SMN2 copies, 0.88 (range: 0.08–5.75) years with 3 SMN2 copies, and 1.0 (range: 0.42–9.5) years with 4 SMN2 copies, which showed a significant difference (*p* < 0.0001).

### Motor functional analyses

**Figure 3:**
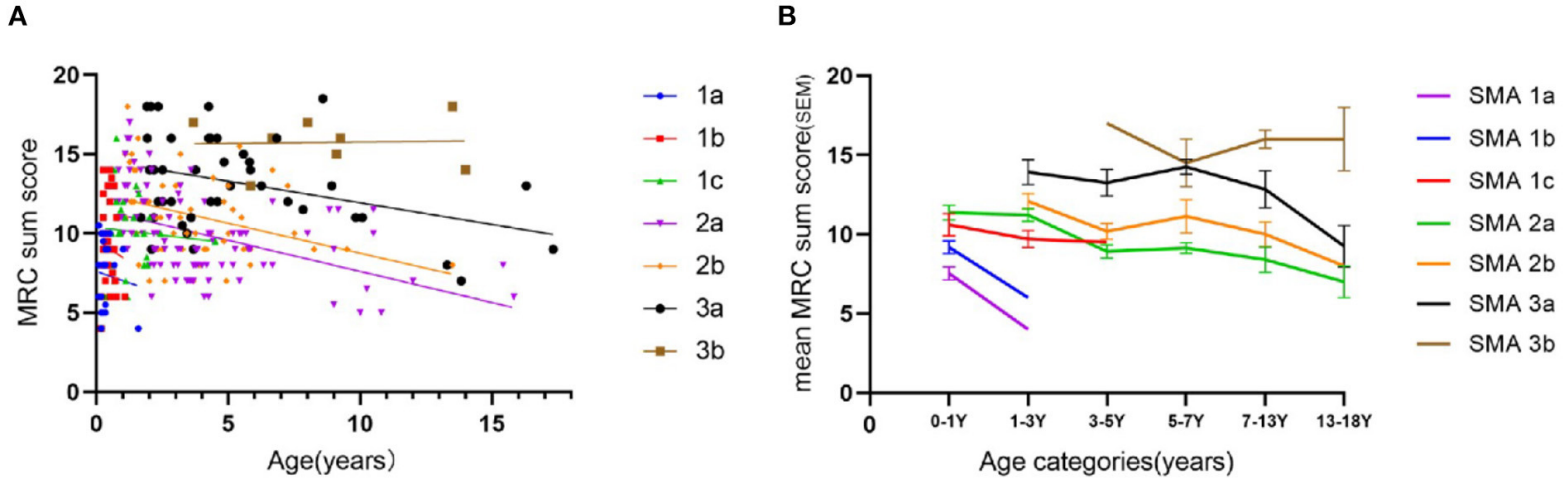
MRC sum scores **(A)** and deterioration pattern of MRC sum scores **(B)** in different subtypes of SMA.

We analyzed 301 measurements of muscle strength (MRC) from 277 patients with SMA 1a−3b (253 patients were evaluated once at their first visits, ranging from 0.08 to 17.33 years old, while 24 patients were reevaluated during their followed ups, ranging from 2.17 to 9.17 years). MRC sum scores were higher in late-onset SMA patients than in early-onset SMA patients, which showed a significant difference (*p* < 0.0001), with a negative linear correlation between scores and ages of assessment in SMA 2a (*r* = −0.5342, *p* < 0.0001) and SMA 2b (*r* = −0.4377, *p* = 0.0005) (Figure 3A). We used linear regression to estimate the rate of decline in the MRC sum score over time and divided patients into age cohorts to estimate the age-specific decline in muscle strength, stratifying for SMA subtypes. There were clear differences in the trend line slopes of the MRC sum score between age cohorts, irrespective of subtype (Figure 3B).

The median age of acquirement of independent sitting was 7.0 (range 6–30) months among SMA 2a patients and 7.0 (range 5–18) months among SMA 2b patients, with no significant difference between subtypes 2a and 2b (*p* = 0.1155). The median age of gain of independent walking was 15 (range 11–66) months in SMA 3a patients and 14 (range 12–24) months in SMA 3b patients, with no significant difference (*p* = 0.4046).

### Event-free survival analysis in SMA type 1 patients

**Figure 4:**
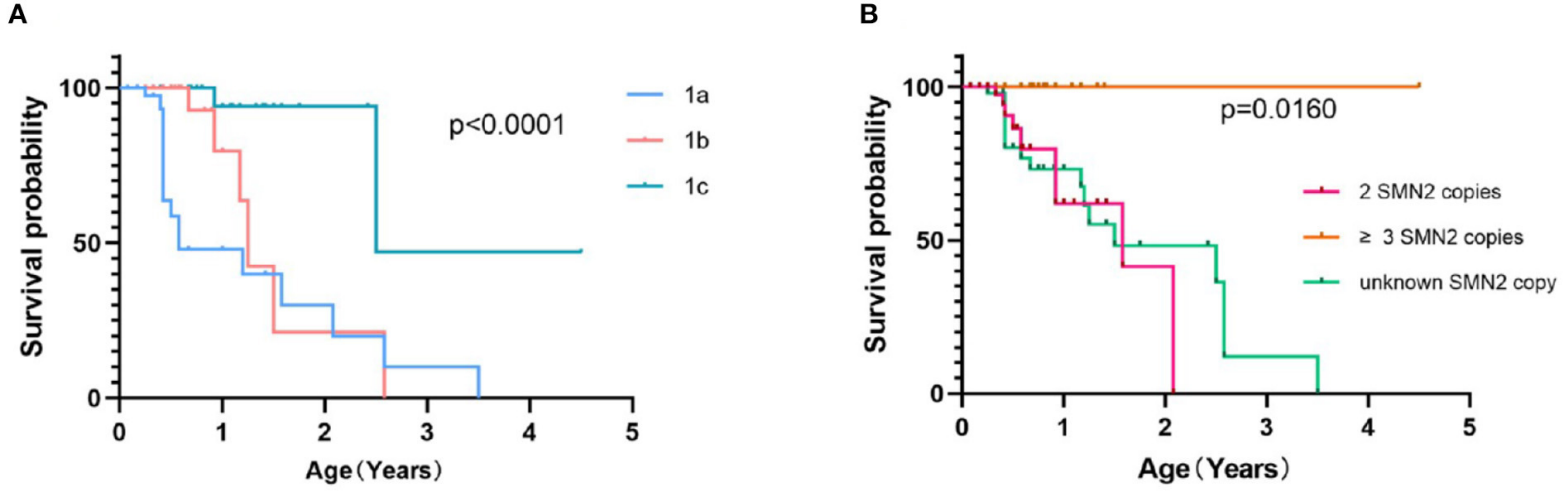
Event-free survival analysis of SMA 1a−1c patients **(A)** and SMA type 1 patients with different SMN2 copy numbers **(B)**.

Survival was evaluated by the Kaplan–Meier method, using death and persistent ventilation (>16 h/days for more than 2 weeks or tracheotomy) as the end of the event. Survival plots of patients with SMA 1a−1c (Figure 4A) showed that the median survival time was 7 months in SMA 1a, and survival probabilities were 48.5% at 1 year and 31.7% at 2 years. The median survival time was 15 months for patients with SMA1b, and survival probabilities were 79.8% at 1 year and 21.0% at 2 years. The median survival time was indeterminate for patients with SMA1c but longer than 2.51 years, and survival probabilities were 94.1, 93.6, and 47.0% at 1, 2, and 3 years, respectively. Kaplan–Meier survival plots of patients with SMA type 1 with different SMN2 copies (Figure 4B) showed that the median survival time was 1.58 years in patients with 2 SMN2 copies, undefined in patients with ≥3 SMN2 copies, and 2.08 years in patients with unknown SMN2 copies (*p* = 0.0160). All patients with SMA 2b, 3a, and 3b were alive, aged from 13 months to 14 years old.

### Survival analysis of time to lose motor milestones in SMA patients types 2 and 3

**Figure 5:**
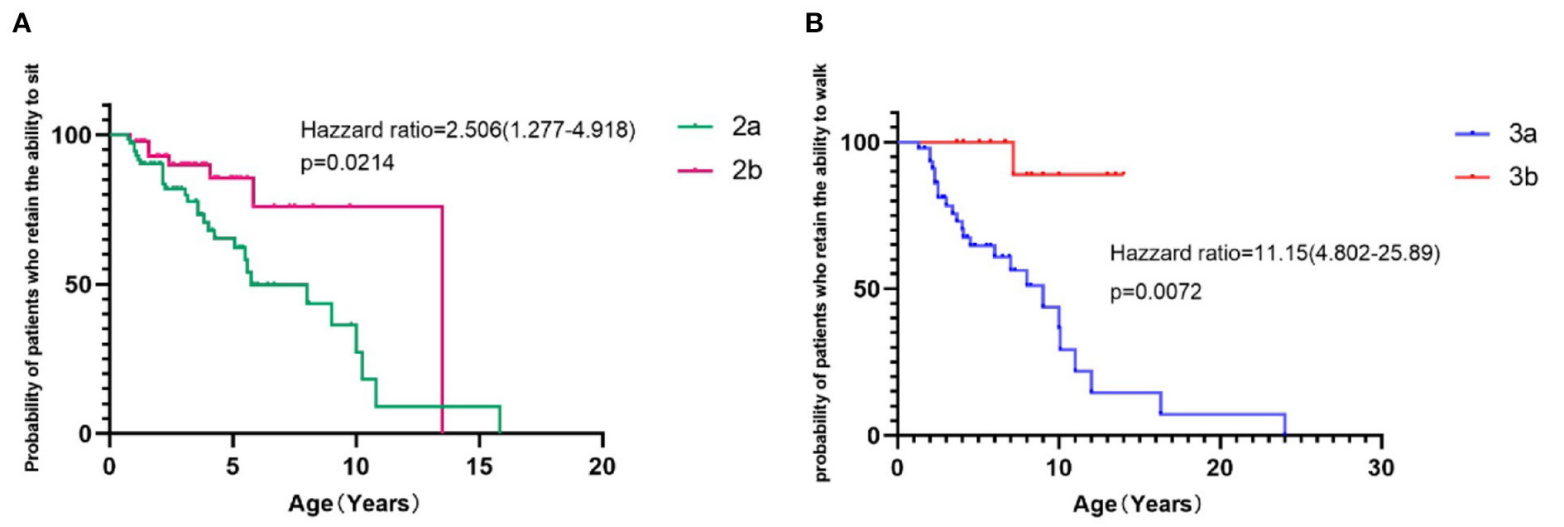
Survival analysis of time to lose the ability of independent sitting and walking in SMA 2a−3b patients. The hazard ratios indicate the risk of loss of sitting in SMA 2a compared with SMA 2b patients **(A)** and the risk of loss of ambulation in SMA 3a compared with SMA 3b patients **(B)**; 95 percent confidence intervals are shown in parentheses. *P*-values were calculated with the use of the stratified log-rank test.

Time to lose the ability to sit and walk was estimated by the Kaplan–Meier method. The median age at which the ability to sit independently (>30 s) was lost was 5.75 years in SMA 2a and 13.5 years in SMA2b (Figure 5A, *p* = 0.0214). The median age of lost ability to walk independently (>10 meters) was 9.0 years in SMA 3a and undefined in SMA3b (Figure 5B, *p* = 0.0072).

**Figure 6:**
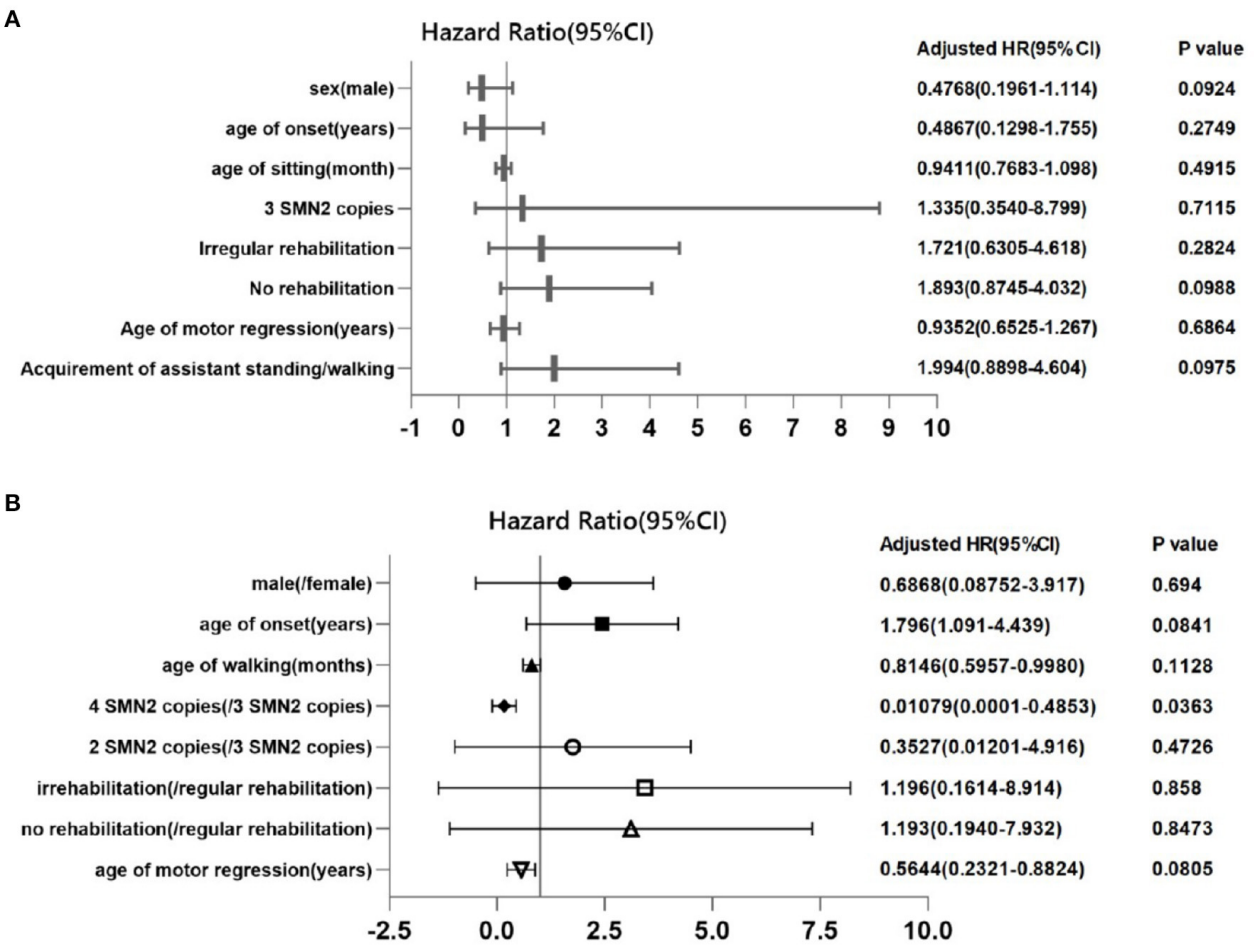
Cox proportional regression of loss of ability to sit independently in SMA 2 patients and to walk independently in SMA 3 patients. Each symbol represents the adjusted hazard ratio of the prognostic factor, and the horizontal lines represent the 95% confidence intervals. The *P*-value for 3 SMN2 copies is for the comparison of patients who had 2 SMN2 copies in SMA type 2 patients **(A)**, and the *P*-value for 4 SMN2 copies is for the comparison of patients who had 3 SMN2 copies in SMA type 3 patients **(B)**.

We also evaluated the prognostic factors of loss of ability to sit in SMA 2 (Figure 6A) and loss of ambulation in SMA 3 patients (Figure 6B) by multiple factor Cox regression analysis, which showed that higher (four) SMN2 copies (HR 0.01079, 95% CI: 0.0001–0.4853) were protective factors for remaining of ambulance in SMA 3 patients.

### Orthopedic deformity and treatment

**Figure 7:**
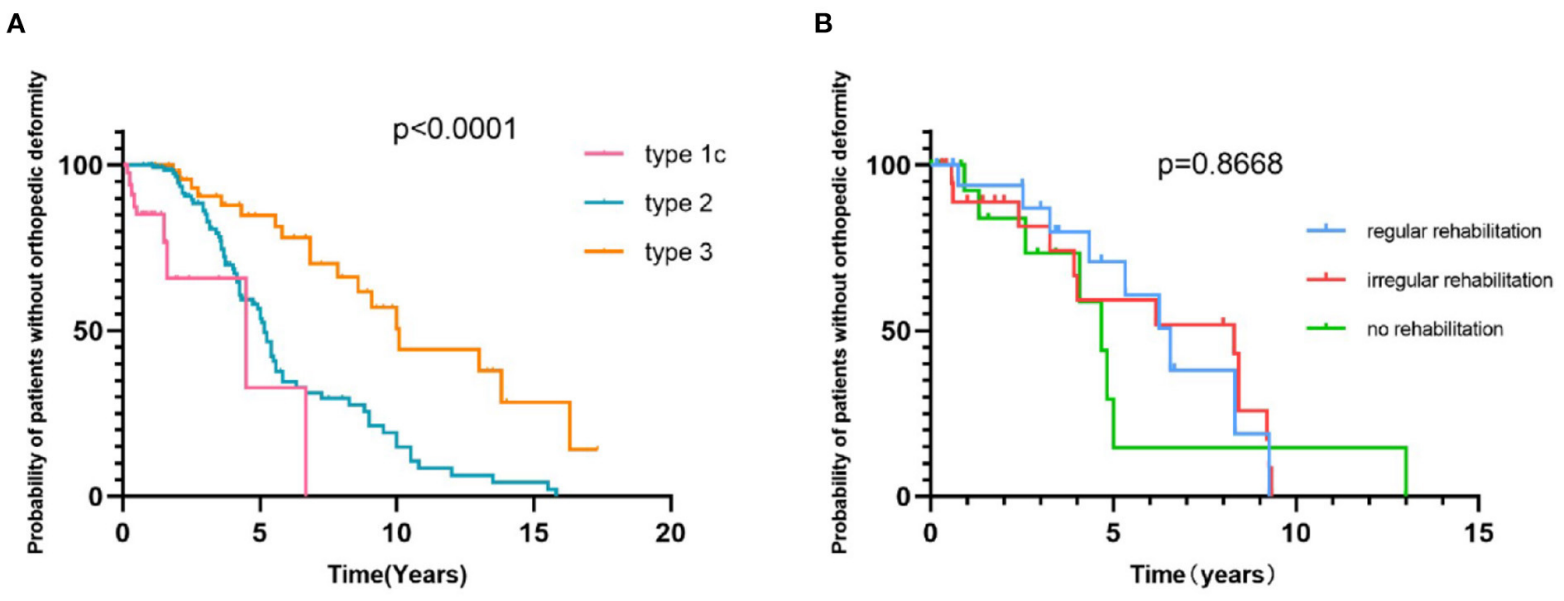
Survival analysis of time from onset to the development of orthopedic deformities in SMA 1c−3b patients **(A)** and in SMA patients with or without rehabilitation **(B)**.

In our cohort, the proportion of regular rehabilitation in SMA patients with types 1a, 1b, 1c, 2a, 2b, 3a, and 3b was 0, 11.3, 12.9, 33.3, 29.1, 21.2, and 21.4%, respectively. Orthopedic deformity data were available for 285 SMA patients, with a follow-up time of up to 15.8 years and a prevalence of 35.1% with single or multiple orthopedic deformities. The most common orthopedic deformity was ankle joint contracture (77/285, 27.0), followed by knee joint contracture (52/285, 18.2%) and spine deformity (28/285, 9.8%). Furthermore, we analyzed the status of orthopedic deformities in the duration of the disease course among different types of SMA and SMN2 copy numbers, using time to progress to orthopedic deformities. The median time from onset to skeletal deformity in SMA with type 1c, type 2, and type 3 was 4.5, 5.2, and 10.1 years, respectively (Figure 7A, *p* < 0.0001). The median time to progress of skeletal deformity was 6.55, 8.3, and 4.67 years for patients with regular, irregular or no rehabilitation, respectively, without a significant difference (Figure 7B, *p* = 0.8668). No orthopedic treatment was performed in any SMA patients.

### Recurrent respiratory infection and usage of mechanical ventilation

**Figure 8:**
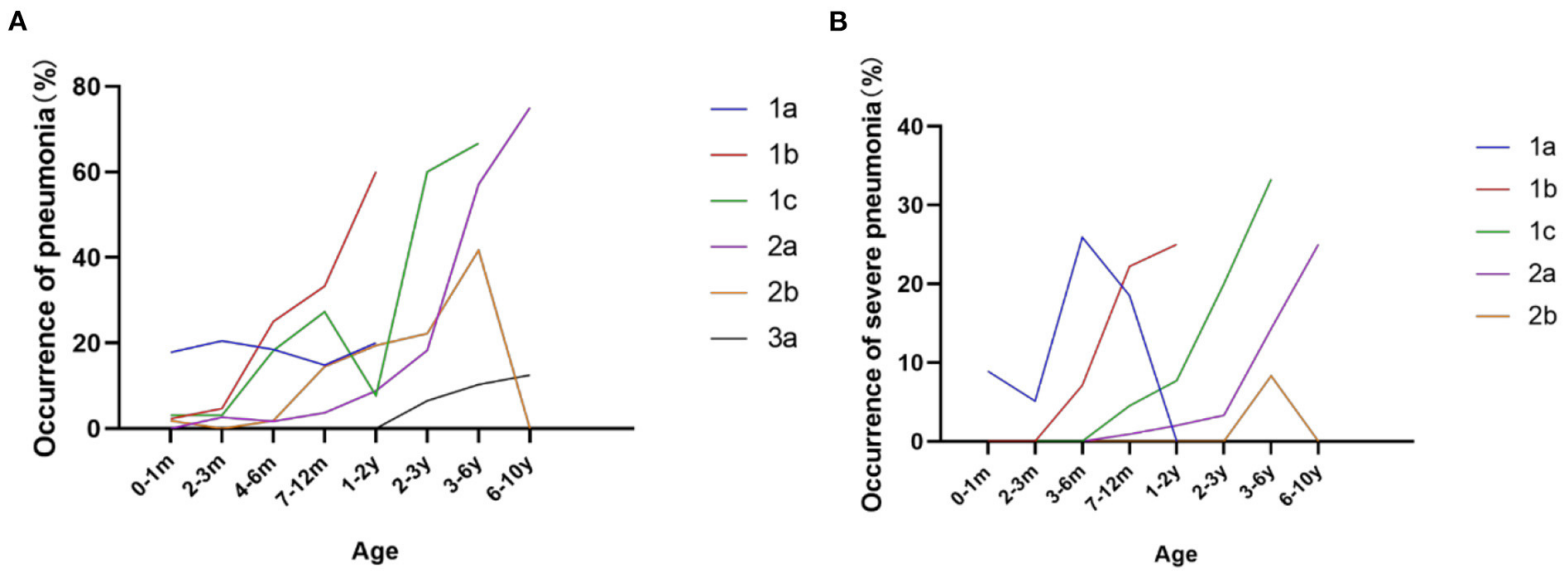
Frequency of pneumonia **(A)** and severe pneumonia **(B)** during different ages in SMA 1a−3a patients.

Respiratory complications, such as recurrent respiratory tract infection and hypoventilation, are the most important causes of morbidity and mortality in SMA. Compared to the high frequency of pneumonia and assistant ventilation before 2 years old in SMA 1a and 1b patients, SMA 1c and 2a patients were at a high risk of pneumonia and assistant ventilation between 2 and 6 years old (Figure 8). From our study, the probability of recurrent pneumonia during infancy (≥3 times/year) also varied among subtypes of SMA 1 patients, which accounted for 40.7, 22.2, and 0% in SMA 1a, 1b, and 1c, respectively. In addition, 7.4 and 5.6% of type 1a and 1b patients, respectively, suffered from recurrent severe pneumonia (mechanical ventilation or intensive care unit admission required) during infancy. Assistant ventilation was required at least once in 8 (17.8%), 4 (9.1%), 3 (9.3%), 7 (6.0%), and 1 (1.8%) patients out of all the type 1a, 1b, 1c, 2a, and 2b patients during the disease course, respectively (Table 1).

### Feeding/swallowing problem

In our cohort, 45/121 (37.2%) SMA type 1 patients had feeding difficulty at first assessment or follow-ups, and 36/177 (20.3%) SMA 2 and 6/66 (9.1%) SMA 3 patients had feeding problems (Table 1). However, only 4/45 (8.9%) patients received nasogastric tube support, and 2/45 (4.4%) patients received gastrostomy.

## Discussion

Since the introduction of SMN2-modifying therapies such as nusinersen and risdiplam, the great importance of SMN2 copy numbers has been widely realized and tested in suspected SMA patients recently in China. The proportion of patients with heterozygous deletion combined with point mutation in our study is lower than previous reports (1, 2), which was likely due to the limitation of molecular tests in China and some missing patients. From the distribution of SMN2 copies among different SMA clinical phenotypes, a generally higher portion of more SMN2 copies was observed in milder SMA phenotypes, which was mainly consistent with the widespread view that an increased SMN2 copy number alleviates the onset and clinical course of SMA (17, 18). However, 2 SMN2 copies were observed across the broad spectrum from the severest SMA 1a to the mildest 3b, seemingly not always predictive of a severe phenotype, as already reported in previous papers (19). This finding implies that other important underlying factors can modify the phenotypes of patients even with few SMN2 copies (3). Further investigation is needed, and new treatment is possible.

From the trajectories of motor deterioration, SMA 1a and 1b patients presented relatively similar, while 1c patients presented much differently from SMA 1a−1b, with a slower decline as well as longer survival time being worth noting, which was consistent with previous reports (20, 21). The inclusion of head control in the criteria for defining 1c is probably because the achievement of higher motor function in 1c patients with known (such as SMN2 compensation) or underlying unknown pathophysiological mechanisms supplies a higher platform for subsequent motor deterioration. As there is still no full consensus on the more recent classification in types 1a, b, and c (22), it is necessary to differentiate 1c from 1a and 1b patients, especially during the assessment of the efficacy of new treatments. Among SMA type 1 patients, the median event-free survival time with 2 SMN2 copies was much worse than that with ≥3 SMN2 copies (1.58 years vs. undefined, *p* < 0.05), consistent with the main opinion that more SMN2 copies were still important protective survival factors (7, 12).

The motor function of SMA 2a, 2b, and 3a all revealed a plateau at ~5–7 years old, which was mainly consistent with a previous report (23, 24), while SMA 3b patients showed a relatively stable period or even “improvement” between 7 and 13 years (25). Thus, the explanation of the efficacy of new therapies during these periods should always be very careful, and the long-term motor trajectory of SMA 2 and 3 patients is necessary. Our study also provided insight into the pattern referring to the gain and loss of motor milestones in subtypes of pediatric SMA patients, which was an important supplement to late-onset and adult patients (26). The median age of ambulation loss was 9.0 years in SMA 3a and undefined in SMA3b (*p* < 0.05), slightly different from a report pointing out that the mean age of ambulation loss was 11.47 years for the type 3a and 13.43 years for the type 3b patients (25). This difference is possibly because of less frequent rehabilitation and other standard care performed in SMA 3a patients in China, and the sample of SMA 3b patients was too small, which requires to enlarge the sample size of SMA 3b and do further investigation. The correlation between SMN2 copies and prognosis of SMA types 2 and 3 remains controversial (25). In our study, Cox regression analysis indicated that compared to 2 SMN2 copies, 3 SMN2 copies were not correlated with milder deterioration of loss of sitting in SMA 2 patients. In contrast, in SMA 3 patients, 4 SMN2 copies implicated better remaining of ambulance, compared to 3 SMN2 copies.

Respiratory complications are one of the most serious risks of mortality in SMA type 1 patients (27). We found that the peak time of severe respiratory complications (hospitalization required) was 3–6 months in SMA 1a, 7–12 months in SMA 1b, and 1–3 years in SMA 1c, mimicking one report from Argentinian in 2020 (28), which was mainly consistent with the survival situation in SMA type 1 patients. A high proportion of severe respiratory complications was observed; however, an assistant coughing machine and ventilation were not widely accepted in SMA type 1 patients.

The development of standards of care for children with SMA had to some degree changed the natural history. However, the situation of compliance standards of care in the SMA population of China is still unsatisfactory (29). A pilot study comparing a natural group of SMA 1 patients with a palliative group with health care in China pointed out that active health management improves the survival rate of SMA patients, reduces the incidence of complications, and improves the prognosis of patients (30). However, respiratory and feeding support, which is critical in SMA type 1 patients, is not as widely accepted as in Western countries. Some patients are seen only once after diagnosis and probably just taken home. Only a very small portion of SMA patients insisted on regular rehabilitation (7.4, 31.1, and 21.2% for SMA types 1, 2, and 3, respectively). In our cohort, none had received orthopedic surgery despite the high proportion of patients with multiple severe orthopedic deformities, which was partly due to the consideration of palliative, limited benefit and risk of complications. Based on our data, rehabilitation, whether regular or irregular, was helpful for delaying the occurrence of orthopedic deformities, even though there was no significant difference. With the availability of advanced drug therapy, standard care of SMA, including regular rehabilitation, respiratory assessment and feeding support, is becoming increasingly prevalent not only among clinicians but also SMA patients in China.

One of the main limitations of our study is the lack of data on motor scale assessments, such as the Hammersmith Functional Motor Scale Expanded (HFMSE) (31) and the Children's Hospital of Philadelphia Infant Test of Neuromuscular Disorders (CHOP INTEND), none of which had been widely used until the introduction of new treatments. A prospective study with more detailed and standard data in treatment-naïve patients with SMA in our center is ongoing.

## Conclusion

Our study included a large cohort of SMA patients from infants with neonatal onset to a milder phenotype of type 3b and elucidated an overall profile and trajectory history of pediatric SMA in China. The cumulative findings will be of great use for comparison to real-world patients treated with recently approved therapies that have shown encouraging results in clinical trials.

## Data availability statement

The raw data supporting the conclusions of this article will be made available by the authors, without undue reservation.

## Ethics statement

Ethics approval for the study was obtained from the Ethical Committee of Children's Hospital of Fudan University. Participant consent was waived by the Ethical Committee of Children's Hospital of Fudan University, as this was a retrospective observational study, and no identifying individual data were used for reporting. All methods were carried out in accordance with relevant guidelines and regulations.

## Author contributions

CH prepared and drafted this manuscript. YS conducted the electromyogram tests. XZ, WL, and LZ helped in the acquisition of medical history. SZ helped reviewing and analysis of the data. XL fulfilled the clinical genetic diagnosis and revised the manuscript. YW designed the study and approved the submission. All authors contributed to the article and approved the submitted version.
